# Smoking among healthcare workers: prevalence, knowledge and attitudes

**DOI:** 10.1192/j.eurpsy.2026.10505

**Published:** 2026-07-31

**Authors:** H. Ziedi, N. Chaouch, S. Kamoun, S. Mihoub, S. Bey, G. Amri, R. Ghachem, N. Mechergui

**Affiliations:** 1 occupational Health depatment; 2 cardiology department; 3 Stomatology Department, Habib Thameur Hospital, Tunis; 4 Department B of Psychiatry, Razi Hospital, Mannouba, Tunisia

## Abstract

**Introduction:**

Smoking remains a significant public health concern, particularly among healthcare workers, who are expected to promote health and wellbeing. The prevalence of smoking among healthcare professionals, their knowledge of smoking-related risks, and their attitudes towards smoking cessation in hospital settings are crucial factors in understanding the broader implications of tobacco use in healthcare environments.

**Objectives:**

The aim of this study was to assess the prevalence of smoking among healthcare workers at Habib Thameur Hospital in Tunis, examine their knowledge about the dangers of smoking, and evaluate their attitudes towards smoking-related policies and smoking cessation efforts within the hospital.

**Methods:**

This is a descriptive cross-sectional study conducted at Habib Thameur Hospital in Tunis in july 2025 involving all healthcare personnel. Data were collected using a pre-established questionnaire including socio-professional data, data on smoking behaviour and an assessment of nicotine dependence.

**Results:**

We surveyed 111 healthcare staff with a mean age of 41.5±11.4 years, and a female predominance of 71.2%. The most represented professional category was paramedical staff (61.8%). Regarding knowledge about smoking, 47.7% believed that cigarette smoke poses the same danger to smokers and non-smokers, and 41.8% were unaware of the availability of smoking cessation consultation at the hospital. Half of those interviewed believed that a designated smoking area should be provided in hospitals, and 84.5% strongly agreed with the ban on smoking in hospitals. The survey revealed that 19.3% of healthcare professionals were active smokers, 2.8% were ex-smokers, and 35.8% were passive smokers. The mean age of onset of smoking was 19±4 years. The majority of smokers consumed an average of 15±5 light cigarettes per day. In terms of nicotine dependence, 33.3% of healthcare workers smoked their first cigarette within an hour of waking up. Regarding attitudes, 38.1% of healthcare workers smoked in their department in the presence of patients. Smokers tried to quit smoking in 76.2% of cases, and 95.2% wanted to quit in the future, with 33.3% reporting the need for psychological support. Passive smokers are exposed to tobacco smoke at work (61.7%), at home (23.4%), and both (14.9%). They leave the area without asking the smoker to stop in 73.3% of cases.

**Conclusions:**

This study highlights a concerning prevalence of smoking among healthcare workers, despite their knowledge of the risks associated with tobacco use. It also underscores the need for stronger smoking cessation support and stricter enforcement of no-smoking policies within healthcare facilities. Given the influence of healthcare professionals on patient behavior, addressing smoking habits and attitudes among these workers is crucial for promoting a tobacco-free healthcare environment and setting an example for the public.

**Disclosure of Interest:**

None Declared

